# Case report: tumefactive demyelinating lesions after the second cycle of alemtuzumab in multiple sclerosis; immune cell profile and biomarkers

**DOI:** 10.3389/fimmu.2024.1395749

**Published:** 2024-07-03

**Authors:** Neus Rabaneda-Lombarte, Aina Teniente-Serra, Anna Massuet-Vilamajó, Cristina Ramo-Tello, Silvia Presas-Rodríguez

**Affiliations:** ^1^ Neurosciences Department, Hospital Universitari Germans Trias i Pujol, Badalona, Spain; ^2^ Immunology Division, Clinical Laboratory MetroNord (LCMN), Hospital Universitari Germans Trias i Pujol and Research Institute (IGTP), Badalona, Spain; ^3^ Department of Cell Biology, Physiology and Immunology, Universitat Autònoma de Barcelona, Badalona, Spain; ^4^ Radiology Service, Hospital Universitari Germans Trias i Pujol, Badalona, Spain

**Keywords:** alemtuzumab, multiple sclerosis, tumefactive demyelinating lesions, rebound, immune subpopulations, biomarkers, case report

## Abstract

**Objective:**

We present a case of multiple tumefactive demyelinating lesions (TDLs) emerging 24 months after the second cycle of alemtuzumab treatment.

**Methods:**

A woman with relapsing-remitting multiple sclerosis (MS) discontinued fingolimod treatment due to gestational desire, which resulted in a severe disease exacerbation. Alemtuzumab was initiated, accompanied by regular clinical, radiological, and immunological monitoring.

**Results:**

She relapsed prior to the second cycle, exhibiting 12 T1Gd^+^ lesions, and peripheral blood showed an increase in B-cells and a decrease in T-cells. At 24 months following the second cycle, she developed cognitive impairment and multiple T1Gd^+^ lesions, including TDLs, were evident on the brain MRI. We found not only an increase in B-cells but also in Th1 central memory cells. Th1/Th17 cells increased 3 months before the detection of TDLs.

**Conclusions:**

TDLs can appear 24 months after the second cycle of alemtuzumab treatment in MS. The increase in Th1/Th17 cells could be a candidate biomarker for TDLs in alemtuzumab-treated MS patients.

## 1 Introduction

Alemtuzumab, a highly effective anti-CD52 monoclonal antibody for relapsing-remitting multiple sclerosis (RRMS), acts mainly through the depletion and subsequent repopulation of T and B lymphocytes. It is the first induction therapy approved for MS, enabling disease remission for years and facilitating pregnancy planning (1).

We report a patient who, 24 months after the second cycle of alemtuzumab, experienced a rebound phenomenon of MS with the appearance of multiple tumefactive demyelinating lesions (TDLs). We describe the variations in immune subpopulations that underline this phenomenon, thus providing insight into the pathophysiological mechanisms.

## 2 Case description

A woman in her 30s was diagnosed with RRMS at the age of 24 following optic neuritis and cervical myelitis. Brain and spinal cord MRI showed typical demyelinating lesions, two of which featured contrast enhancement (Gd^+^). Analyses of cerebrospinal fluid (CSF) revealed the presence of oligoclonal IgG bands (OGB). Interferon-beta treatment was initiated (Expanded Disability Status Scale (EDSS) 2.0), but it was switched to fingolimod (Gilenya^®^) 2 years later due to persistent relapses that led to increased disability (EDSS 6.0).

After 2 years of disease stability (EDSS 5.5), fingolimod was discontinued due to a desire for pregnancy. Two months later, she experienced severe disease activation with spinal cord lesions (EDSS 7.0), requiring methylprednisolone (MP) and plasma exchange (EDSS 6.0). Alemtuzumab was initiated after a 4-month washout period from fingolimod, and her plans for pregnancy were deferred.

Neurological evaluations and blood analysis were conducted every 3 months, along with annual MRIs. Immune monitoring using flow cytometry in whole blood samples was carried out prior to beginning treatment with alemtuzumab and monthly during the follow-up. The temporal profiles of different lymphocyte subpopulations, including B lymphocytes (naive, transitional, class-switched and unswitched memory cells), T lymphocytes (naive, central memory, effector memory and EMRA cells, as well as regulatory T lymphocytes, Th1, Th2, Th17 and Th1/Th17 cells), and natural killer cells, were analyzed.

**Figure 1:**
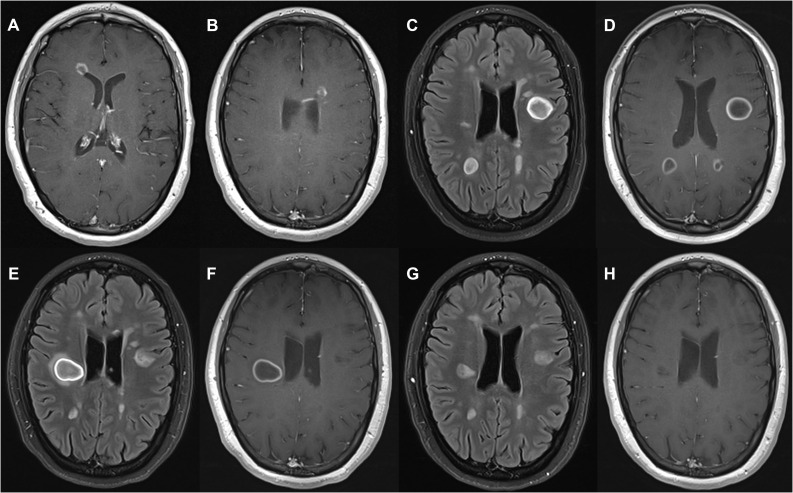
Radiological findings in a patient with RRMS after treatment with alemtuzumab. **(A, B)** Brain MRI obtained 11 months after the first cycle. Post-contrast T1 images showed new gadolinium-enhancing lesions, some of them with open-ring enhancement. **(C, D)** Brain MRI conducted 24 months after the second cycle revealed several new demyelinating lesions on FLAIR images **(C)**, some of them showing large morphology with closed-ring enhancement located in the left frontal opercular juxtacortical white matter (maximum diameter 21 mm) and bilateral parietal subcortical white matter **(D)**. **(E, F)** Brain MRI conducted 2 months later revealed an increased size of the lesion in right corona radiata (maximum diameter 25 mm) with closed-ring enhancement, perilesional edema and mild mass effect on the right lateral ventricle, and also a reduction in the size of the left frontal and bilateral parietal lesions with resolution of contrast enhancement. **(G, H)** After 3 months of natalizumab treatment, brain MRI illustrated a remarkable reduction in lesion size on FLAIR images **(G)** with complete resolution of the contrast enhancement **(H)**. MRI: Magnetic resonance imaging, RRMS: relapsing-remitting multiple sclerosis. FLAIR: Fluid-attenuated inversion recovery.

**Figure 2:**
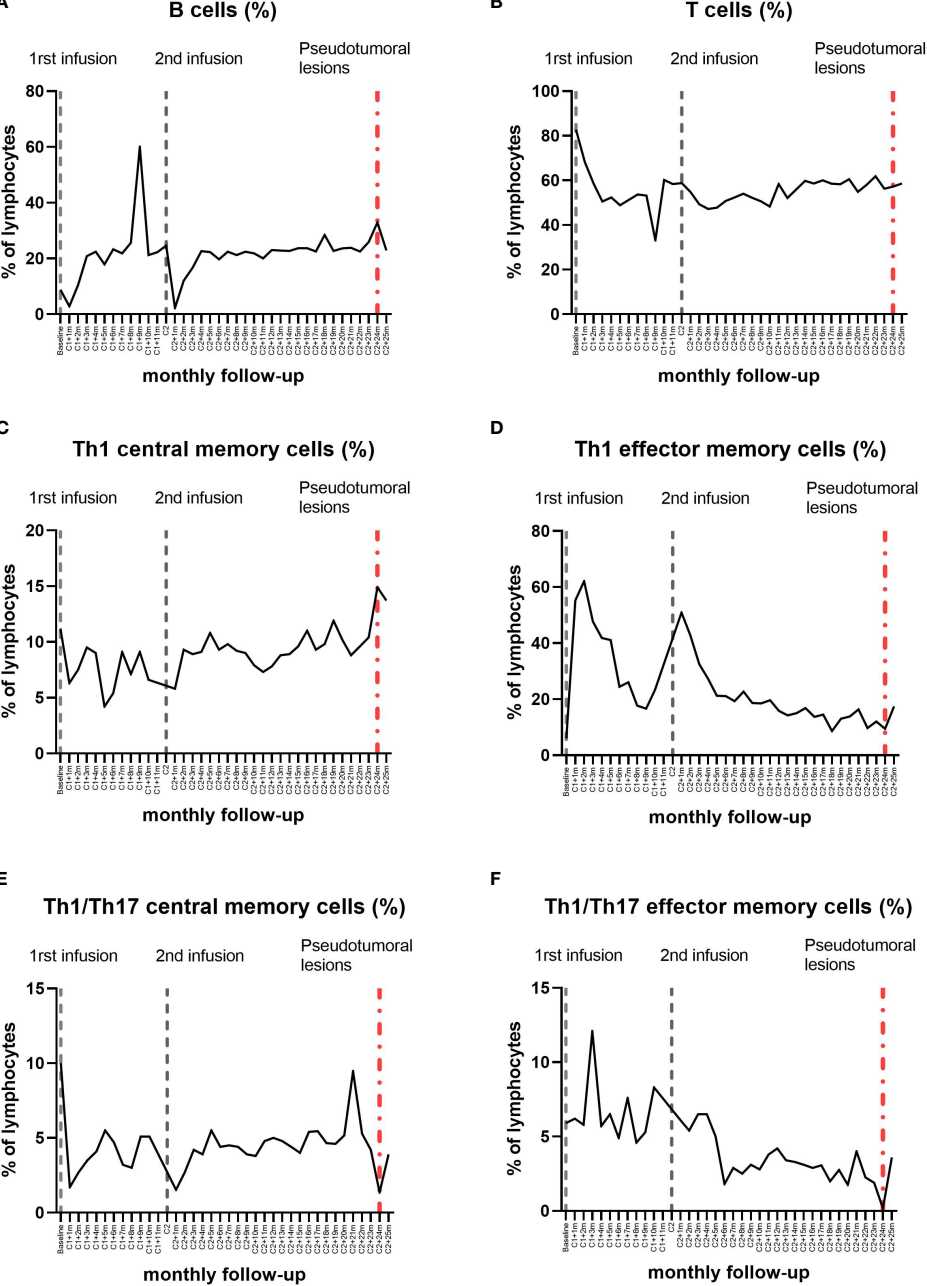
Longitudinal dynamics in the percentage of the lymphocyte subpopulations in the peripheral blood by flow cytometry during alemtuzumab treatment in the patient. Between 8 and 10 months after the first cycle of alemtuzumab, at the same time as the mild sensory event and the multiple gadolinium-enhancing Gd^+^ lesions appearance, we observed an increase in the percentage of total B lymphocytes **(A)** and a decrease in the percentage of total T lymphocytes **(B)** in peripheral blood. Together with the appearance of the tumefactive demyelinating lesions, we observed an increase in the percentage of total B lymphocytes **(A)** and, regarding the Th1 memory cells **(C, D)**, an increase in the percentage of Th1 central memory cells **(C)**. Three months before, an increase in the percentage of Th1/Th17 cells was observed **(E, F)**.

**Figure 3:**
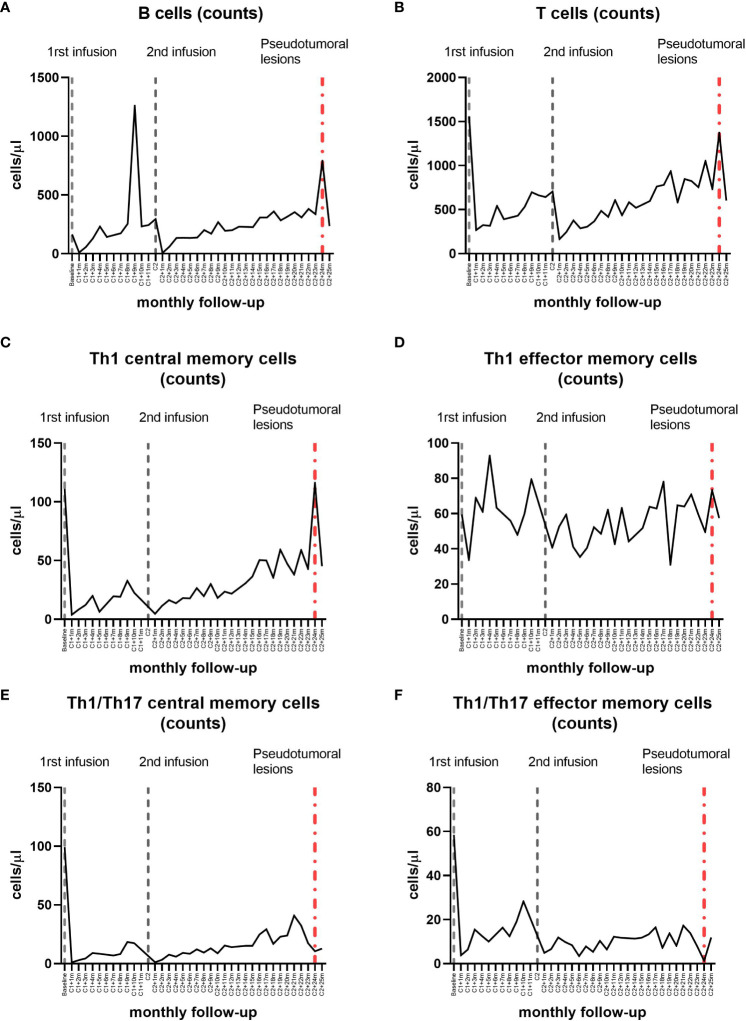
Longitudinal dynamics in absolute counts of lymphocyte subpopulations in the peripheral blood by flow cytometry during alemtuzumab treatment in the patient. Between 8 and 10 months after the first cycle of alemtuzumab, at the same time as the mild sensory event and the multiple gadolinium-enhancing Gd^+^ lesions appearance, we observed an increase in the absolute counts of total B lymphocytes **(A)** in peripheral blood. Together with the appearance of the tumefactive demyelinating lesions, we observed an increase in the absolute counts of total B and T lymphocytes **(A, B)** and an increase in the absolute counts of Th1 memory cells **(C, D)**, specially Th1 central memory cells **(C)**. Three months before, an increase in the absolute counts of Th1/Th17 cells was observed **(E, F)**.

Nine months following the first cycle of alemtuzumab, she underwent a mild sensory relapse, with improvement after MP treatment. A brain MRI conducted 2 months later revealed 12 new T1Gd**^+^** lesions (**Figures 1A, B**). An increase in the percentage and absolute counts of total B-cell populations (**Figures 2A**, **3A**) was detected in peripheral blood at the time of this sensory event, coupled with a decrease in the percentage of total T-cell populations (**Figure 2B**). No changes were noted in minor B- and T- cell subsets.

Six months after the second cycle, there was a progressive clinical improvement, that resulted in autonomous ambulation (EDSS 4.0) and brain MRI results showing inflammation resolution.

Twenty-four months following the second cycle, she developed cognitive impairment and heightened fatigue. The brain MRI revealed multiple new T1Gd**^+^** demyelinating lesions, including multiple TDLs with closed-ring enhancement, perilesional edema and mass effect on the lateral ventricles (**Figures 1C, D**). She underwent MP treatment. An extensive study including anti-AQP4 and anti-MOG antibodies in serum was negative. The CSF analysis carried out through flow cytometry revealed few cells, predominantly T lymphocytes, and the IgG/albumin index and OGB were similar to those at the time of diagnosis. In peripheral blood, we found again a rise in the percentage and absolute counts of B-cells (**Figures 2A**, **3A**), as well as an increase in the absolute counts of T-cells (**Figure 3B**). Regarding Th1 memory cells (**Figures 2C, D**, **3C, D**), an increase in the percentage and absolute counts of Th1 central memory cells was observed (**Figures 2C**, **3C**). Transitional B-cells, defined as CD19^+^CD27^-^CD24^hi^CD38^hi^, were analyzed with no differences. In contrast, an increase in the percentage and absolute counts of Th1/Th17 cells (**Figures 2E, F**, **3E, F**), specially central memory (**Figures 2E**, **3E**), was detected 3 months prior to the TDLs detection.

Given the appearance of new TDLs in a routine MRI scan after 2 months (**Figures 1E, F**), she underwent MP treatment again and natalizumab was initiated (JC virus negative). After 3 months of natalizumab treatment, the size and enhancement of TDLs diminished (**Figures 1G, H**).

## 3 Discussion

The rebound effect in MS refers to disease activity exceeding the level expected based on the pre-treatment period, and it has been widely reported after natalizumab or fingolimod withdrawal. Although the pathogenic mechanism remains elusive, the immunological recovery of lymphocyte subpopulations may be implicated (2).

Alemtuzumab can cause severe autoimmune disorders, attributed to autoantibody production, peripheral T-cell proliferation against self-antigens, and disruption of immune cell balance during reconstitution. This imbalance manifests as rapid B-cell repopulation without effective T-cell regulation (3). Cases of autoimmune encephalitis have been reported, one of them with identification of anti-GABA_A_ receptor antibodies (4).

Literature reports 20 cases of rebound phenomenon following alemtuzumab treatment, also referred to as paradoxical disease activation, exacerbation of the disease, or unexpected high disease activity (5–13). They occurred after the first cycle of treatment (5–13), with one case occurring after both the first cycle and a few months following the second (9). Unlike our patient, all documented cases presented severe symptoms. Only one report described the emergence of a TDL, but unlike our patient’s case, this happened after the first cycle (6). The development of TDLs in neuromyelitis optica after three cycles of alemtuzumab has also been reported (14).

The excessive disease activation after alemtuzumab is associated with an increase in B-cells, along with a reduction in T-cells and a deficiency of a regulatory B-cell subset (CD19^+^CD24^hi^CD38^hi^ cells). Furthermore, the decrease in this regulatory B-cell subset is suggested to be a predictive biomarker of disease exacerbation following alemtuzumab treatment (9, 15). In line with this, our patient experienced a rebound 9 months after the first cycle; the total B-cells increased in peripheral blood and total T-cells decreased. However, no differences were observed in CD19^+^CD27^-^CD24^hi^CD38^hi^ cells.

Regarding specifically to the emergence of TDLs following alemtuzumab treatment in MS, the only reported case associates the TDLs with an increase in B-cells and a decrease in CD4^+^ and CD8^+^ T-cells in peripheral blood (6). Similarly, the appearance of our patient’s TDLs was associated with an increase in total B-cells in peripheral blood. However, we also observed an increase in Th1 central memory cells at the time of TDLs detection, as well as an elevation in Th1/Th17 cells 3 months prior to their detection.

Considering that Th1/Th17 cells are postulated to play a pro-inflammatory role in the pathogenesis of MS (16), the identification of an increase in these cells in peripheral blood before the detection of TDLs suggests they could also be used as a predictive biomarker for disease rebound. It is important to note that we are analyzing these lymphocyte populations in peripheral blood, not in the central nervous system, where they would later migrate to exert their inflammatory function. However, this association should be deeply analyzed in broader studies.

The strength of this report lies in the exceptional rebound manifestation, the severity of radiological findings, and the timing of the presentation. Its limitations include its nature as a case report.

In conclusion, this is the first case reporting a rebound phenomenon post-alemtuzumab occurring beyond 12 months after the second cycle. Moreover, it was associated with the unexpected emergence of TDLs, which, fortunately, presented with minimal symptoms. It was associated to changes in the dynamics of lymphocyte subpopulations, and Th1/Th17 cells could be a predictive biomarker of disease activation in alemtuzumab-treated MS patients. Further studies are needed to corroborate the hypotheses generated from this clinical case.

## Data availability statement

The original contributions presented in the study are included in the article/supplementary material. Further inquiries can be directed to the corresponding author.

## Ethics statement

Written informed consent was obtained from the individual(s) for the publication of any potentially identifiable images or data included in this article.

## Author contributions

NR-L: Conceptualization, Writing – original draft, Writing – review & editing. AT-S: Formal analysis, Writing – review & editing. AM-V: Writing – review & editing. CR-T: Conceptualization, Writing – review & editing. SP-R: Conceptualization, Writing – original draft, Writing – review & editing.
